# A Comprehensive Review of Neuronal Changes in Diabetics

**DOI:** 10.7759/cureus.19142

**Published:** 2021-10-30

**Authors:** Rudy Luna, Ramya Talanki Manjunatha, Bhaswanth Bollu, Sharan Jhaveri, Chaithanya Avanthika, Nikhil Reddy, Tias Saha, Fenil Gandhi

**Affiliations:** 1 Neurofisiología, Instituto Nacional de Neurologia y Neurocirugia, CDMX, MEX; 2 Graduate Medical Education, Kempegowda Institute of Medical Sciences, Bangalore, IND; 3 Internal Medicine, Narayana Medical College, Nellore, IND; 4 Medicine, Smt NHL MMC, Ahmedabad, IND; 5 Medicine and Surgery; Pediatrics, Karnataka Institute of Medical Sciences, Hubli, IND; 6 Internal Medicine, Kamineni Academy of Medical Science and Research Centre, Hyderabad, IND; 7 Internal Medicine, Diabetic Association Medical College, Faridpur, BGD; 8 Medicine, Shree Krishna Hospital, Anand, IND; 9 Research Project Associate, Memorial Sloan Kettering Cancer Center, New York, USA

**Keywords:** blood glucose control, diabetic neuropathy, cognition, diabetes complications, nervous system, “diabetes mellitus”

## Abstract

There has been an exponential rise in diabetes mellitus (DM) cases on a global scale. Diabetes affects almost every system of the body, and the nervous system is no exception. Although the brain is dependent on glucose, providing it with the energy required for optimal functionality, glucose also plays a key role in the regulation of oxidative stress, cell death, among others, which furthermore contribute to the pathophysiology of neurological disorders. The variety of biochemical processes engaged in this process is only matched by the multitude of clinical consequences resulting from it. The wide-ranging effects on the central and peripheral nervous system include, but are not limited to axonopathies, neurodegenerative diseases, neurovascular diseases, and general cognitive impairment.

All language search was conducted on MEDLINE, COCHRANE, EMBASE, and GOOGLE SCHOLAR till September 2021. The following search strings and Medical Subject Headings (MeSH terms) were used: “Diabetes Mellitus,” “CNS,” “Diabetic Neuropathy,” and “Insulin.” We explored the literature on diabetic neuropathy, covering its epidemiology, pathophysiology with the respective molecular pathways, clinical consequences with a special focus on the central nervous system and finally, measures to prevent and treat neuronal changes.

Diabetes is slowly becoming an epidemic, rapidly increasing the clinical burden on account of its wide-ranging complications. This review focuses on the neuronal changes occurring in diabetes such as the impact of hyperglycemia on brain function and structure, its association with various neurological disorders, and a few diabetes-induced peripheral neuropathic changes. It is an attempt to summarize the relevant literature about neuronal consequences of DM as treatment options available today are mostly focused on achieving better glycemic control; further research on novel treatment options to prevent or delay the progression of neuronal changes is still needed.

## Introduction and background

Diabetes mellitus (DM) is a metabolic disease characterized by inadequate control of blood glucose levels, mainly a chronic state of hyperglycemia, as well as frequent episodes of hypoglycemia, due to different pathogenic processes, which determine the classification of this disease largely as type 1 and type 2, but there are other specific types attributable to endocrinopathies, drugs, infections, immunologic, genetic, and pancreatic causes. These metabolic dysregulations can lead to multiple complications affecting the heart, kidneys, blood vessels, eyes, and nervous system impacting the quality of life and being the main reason for mortality [1-5].

The brain comprises 2% of our total body weight, consumes 25% of oxygen and around 20% of the body's glucose levels which are regulated primarily by the integration of the hypothalamus with multiple hormones that modulate food intake, energy expenditure, insulin secretion, hepatic glucose production, and glucose/fatty acid metabolism in adipose tissue and skeletal muscle [6-9]. Although the brain is dependent on glucose for providing it with the energy required for optimal functionality from cellular maintenance to neurotransmitter generation. Glucose plays a key role in the regulation of oxidative stress, cell death, and pathways whose mechanisms are implicated in disrupted hypothalamic circuits and sensing of glucose and insulin, which furthermore contribute to the pathophysiology of neurological disorders [10-13]. Therefore, glucose regulation is critical.

Nervous system complications of DM include axonopathies, neurodegenerative diseases, neurovascular diseases, and general cognitive impairment. Not to mention that almost all patients with DM have multiple vascular, metabolic, and other comorbidities that together with uncontrolled glucose levels accelerate neurological complications [14]. Although these effects are well known, the cellular mechanisms, such as increased advanced glycation end (AGE) products, enhanced polyol pathway, neuromodulatory, neurotrophic, microvascular are among other molecular changes which have not been fully understood [15-16].

In this review, we present how the nervous system can be modified by the consequences of DM from molecular to anatomical changes through the central nervous system (CNS), peripheral nervous system (PNS), and autonomic nervous system (ANS) and what clinical effects these changes could have along with short- and long-term complications, risk factors, and a way to prevent or treat it in addition to maintaining normal glucose levels. Our goal is to summarize how uncontrolled glucose levels can be detrimental to the brain anatomically and physiologically and what consequences they may present along with how best to prevent and treat them and hopefully open the door to further research.

## Review

Epidemiology and risk factors

Diabetes mellitus is highly prevalent globally and has caused a significant number of deaths among the population [17]. There has been an exponential rise in DM cases from 108 million to 422 million cases on a global scale [18]. Greater than 8.5% of adults are suffering from diabetes globally causing more than 1.5 million deaths annually [19]. The highest burden of DM is in developing countries especially due to their diets and sedentary lifestyles [20]. Studies have indicated a strong positive correlation between obesity and DM at a global level and most obese individuals develop diabetes [21]. The factors attributable to DM are usually determined by the type of diabetes that the individual is suffering from [22]. Type 1 DM has a strong genetic predisposition and the genes implicated are transmitted down the family line [23]. It is usually characterized by a reduced secretion of insulin eventually resulting in impaired glycemic control [24]. Additionally, type 2 DM is usually shaped by the individuals' lifestyle and diet. Individuals who live a sedentary lifestyle end up becoming obese and eventually develop DM [25]. Understanding such factors and screening the individuals at risk for prediabetic states help prevent the development and progression of diabetes globally [26].

The pathological effects of deranged glucose metabolism are evident in virtually all systems of the body. Without exception, there are significant changes in neuronal bodies [27]. However, not all individuals who have DM tend to develop complications. For instance, diabetic nephropathy tends to develop in individuals with no intervention or management [28]. Thus, poor diabetic control results in glucotoxicity that eventually leads to multiple organ systems complications seen in patients [29]. Therefore, the development of complications is determined by the control of serum glucose levels so those with poorly controlled diabetes are usually at a higher risk [30]. Moreover, age, height, obesity, diastolic blood pressure, smoking status, chronic kidney disease, vascular disease, low HDL cholesterol level, high triglyceride, and glycosylated hemoglobin (HbA1c) levels are associated with a higher risk of neurologic complications in patients with DM especially polyneuropathy [31-34]. Understanding the key factors in pathogenesis allows one to intervene easily and reduce the risk of developing the disease.

Molecular factors damaging neurons

Oxidative stress is a known cause for neuronal changes due to hyperglycemia. Several other molecular factors in major biochemical pathways also have a role to play. Some pathways include the polyol pathway, advanced glycation end (AGE) products pathway, protein kinase C signaling, poly adenosine phosphate ribose polymerase (PARP) pathway, hexosamine pathway, mitogen-activated protein kinase (MAPK) pathway, nuclear factor-κB (NF-kB) signaling, cyclooxygenase pathway, and the role of tumor necrosis factor-α (TNF-α). The roles of the few above-mentioned biochemical pathways in neuronal changes are discussed below [35].

Polyol Pathway

Aldose reductase (AR) and sorbitol dehydrogenase play a major role in the metabolism of glucose through the polyol pathway. AR generally has a lower affinity for glucose. In DM, a hyperglycemic state causes excess glucose to metabolize through the polyol pathway. The first step in the pathway is the reduction of glucose to sorbitol by aldose reductase with the help of nicotinamide adenine dinucleotide phosphate (NADPH) followed by oxidation of sorbitol to fructose by sorbitol dehydrogenase using nicotinamide adenine dinucleotide (NAD)+ [36]. The reduction process leads to an increased sorbitol concentration and depletion of NADPH stores [37]. NADPH is required to produce reduced glutathione (GSH). As the levels of NADPH are depleted there is a downregulation of GSH leading to endothelial cell damage with subsequent loss of nitric oxide-mediated vasodilation impacting negatively the nerve vasculature.

An experiment conducted in transgenic mice with a gain of function mutation in AR activity showed that with increased levels of AR there is a decrease in GSH levels leading to depletion of NADPH which leads to oxidative stress hence correlating with polyol pathway [38]. An increase in sorbitol concentration leads to osmotic stress and efflux of electrolytes in the Schwann cells of peripheral neurons leading to the schwannopathy-related phenotype of diabetic peripheral neuropathy (DPN) [38-39]. The other effects of an increase in intracellular concentration of sorbitol and fructose result in the decrease of myo-inositol and taurine concentration, increase in intracellular Na+, inactivation of Na+/K+ adenosine triphosphatase pump, swelling of axon, axon-glia dysfunction, and reduction of nerve conduction velocity [40]. Nervous tissue differs from other tissues in that it is scarce in the activity of sorbitol dehydrogenase, hence excessive accumulation of sorbitol cannot be managed in this specific tissue leading to the detrimental effects described. This is also the mechanism that explains why specific tissues such as retina or renal tissue are highly impacted by hyperglycemia. The transgenic mouse model showed a delay in motor nerve conduction velocity and nerve fiber atrophy due to polyol accumulation in tissue independent of hyperglycemia [41]. Peripheral nerves with overexpressed AR in Schwann cells showed a greater reduction in motor nerve conduction velocity in transgenic mice with diabetes [42].

Hexosamine Pathway

Due to hyperglycemia, there is an increased concentration of fructose-6-phosphate which proceeds to the hexosamine pathway where it is converted into glucosamine-6-phosphate followed by the production of uridine diphosphate N-acetyl glucosamine (UDPGlcNAc) by glucosamine-6-phosphate amidotransferase. N-acetyl glucosamine (GlcNAc) induces oxidative stress leading to pancreatic beta (β)-cell functional deﬁcit. Overexpression of glutamine fructose-6 phosphate aminotransferase results in inhibition of glucose transporter 2 (GLUT2), insulin leading to hyperglycemia, and increased hydrogen peroxide levels leading to oxidative stress affecting the neuronal environment [43]. Sp1 controls the gene expression of plasminogen activator inhibitor-1 (PAI-1) and transforming growth factor β 1 (TGF-β 1) [44]. Hyperglycemia increases GlcNAc and activation of gene transcription factor Sp1 [45]. Up-regulation of PAI-1 increases vascular smooth muscle cell division which is responsible for atherosclerosis [46].

Advanced Glycation End-Products Pathway

The AGE products are formed by the non-enzymatic reaction of glucose, aldehydes, and other saccharides with nucleotides, proteins, and lipids. Increased concentrations of fructose-lysine and AGE have been found in peripheral nerves of streptozotocin-diabetic rats [47]. N(epsilon)-[carboxymethyl]-lysine an advanced glycation end product is found in increasing concentrations in the sciatic nerves of diabetic rats in contrast to their controls [48]. Schwann cells of peripheral nerves showed the existence of advanced glycation end product receptors (RAGE) and an increase in their concentration in diabetic neuropathy [49]. Advanced glycation end products may cause inflammation and apoptosis through their interaction with RAGE and it also up-regulates NF-κB [50]. RAGE increases the expression of the p65 subunit of NF-κB in diabetic neuropathy [51]. AGE products from glyceraldehyde and glycolaldehyde can cause irreversible death of Schwann cells, decrease in cell replication and viability, up-regulation of NF-κB, mitochondrial membrane potential decrease, and increase in inflammatory cytokines like TNF-α and interleukin-1 β in diabetic rats [52]. The over-activation of NF-κB and its impact on the neuronal function or environment has been explained briefly below.

Oxidative Stress

Oxidative stress occurs whenever there is an imbalance between the free radical scavengers and free radical species (reactive nitrogen, oxygen species) and plays a key role in diabetic neuropathy [53]. Lipids of myelinated structures of nerves may be damaged by reactive free radical species leading to damage of the microvasculature environment of the nervous system [54]. Neuropathic pain occurs as a result of oxidative stress on peripheral nerves leading to hyper-excitability in central neurons and afferent nociceptors causing spontaneous impulses in axon and dorsal nerve ganglia [55]. Nitrosative stress has a key role in diabetic-induced neuropathy [56]. The disparity between GSSG/GSH ratio leads to an increase in lipid peroxidation, decrease in the following enzymes which play a key role in antioxidant cell response such as superoxide dismutase level, ascorbate, and catalase generate oxidative stress in the peripheral nerves [57-58]. Moreover, diabetes-induced oxidative stress can disturb the mitochondrial membrane potential leading to its swelling and permeability [59-60]. 

A study in diabetic rats reported that B cell lymphoma 2 (Bcl-2), an anti-apoptotic protein, is downregulated in dorsal root ganglia leading to disruption of membrane potential in mitochondria and transfer of cytochrome c from the mitochondria to the cytoplasm causing apoptosis through the caspase-3 pathway. Bcl-2 associated X (Bax or Bcl-xL) expression remained the same in diabetic neuropathy. Dynamin-related protein 1 moves from cytosol to mitochondria starting fission and leading to mitoptosis and apoptosis [61]. Peroxisome proliferator-activated receptor-gamma coactivator-1α (PGC-1α) plays a vital role in mitochondrial activity which seems to be downregulated in dorsal root ganglia of diabetic animals. Insulin deficiency along with reactive oxygen species may decrease mitochondrial function in diabetes [62].

Protein Kinase C Pathway

Protein kinase C is a family of serine/threonine-related protein kinases. They affect signal transduction pathways involved with cell differentiation, cell proliferation, and apoptosis. Diacylglycerol, calcium, and phosphatidylserine are required for activation of conventional isoforms (α, β I, β II, and γ) whereas novel isoforms (η, δ, θ and ε) require diacylglycerol for activation and atypical isoforms (ι/λ, M ζ) require neither diacylglycerol nor calcium [63]. Each of the above-mentioned isoforms shows a different activity, distribution, and expression in diabetes. Protein kinase C isoforms α, β I, β II, γ, ε, and δ are detected in nerves when an immunochemical analysis is done [64]. Aldose reductase overexpressing diabetic transgenic mice reported activation of protein kinase c -II β isoform. This protein kinase C upregulates TGF- β 1, vascular endothelial growth factor (VEGF), PAI-1, and NF-κB leading to a variety of diabetic complications [65]. Protein kinase C inhibitor induced normalization of sciatic nerve blood flow and nerve conduction velocity in diabetic neuropathy [66]. Insulin resistance is observed in several studies involving upregulation of protein kinase C in diabetic subjects [67].

Poly Adenosine Phosphate Ribose Polymerase Pathway

Normally, the PARP plays a role in deoxyribonucleic Acid (DNA) repairing and apoptosis. In diabetes subjects, it is observed that overexpression of PARP may cause tissue damage [68]. It has been previously observed that hyperglycemia caused by diabetes leads to the generation of reactive free radicals followed by single-strand DNA breaks and activation of PARP, but more recent studies show that both single-strand DNA breakage and oxidative-nitrosative stress are not required for the activation of PARP rather it occurs due to phosphorylation by extracellular regulated kinase (ERK) [69-70]. Hyperglycemia and nonesterified fatty acids may activate PARP leading to damage of neurilemma through oxidative stress and retinal pericytes [71-72].

Mitogen-Activated Protein Kinase Pathway

The MAPKs relay signal transduction in response to a variety of stimuli. c-Jun N-terminal kinase (JNK), extracellular signal-related kinase (ERK), and p38 are the three families of protein kinases in MAPKs [73]. An ERK 1/2 regulates neural survival. Also, it may be involved in the development of neuropathic pain. JNK and p38 are involved in neural apoptosis [74-76]. Spinal ganglia neurons of streptozotocin-diabetic rats showed an increase in levels of p38, JNK, and ERK [77]. Sural nerves of type 1 and type 2 diabetic patients showed up-regulation of JNK and p38 [78]. Diabetic rats showed improved regeneration in dorsal root ganglia (DRG) neurons on inhibiting persistently activated JNK and p38 activation in DRG of streptozotocin-induced diabetic rats [79-80].

Nuclear Factor-kB Pathway

Nuclear factor-kB (NF-kB) is a transcriptional factor that mediates inflammatory, immune responses, and apoptosis. Inflammatory stimuli activate NF-kB. Dorsal root ganglia, sciatic and sural nerves in diabetic transgenic mice showed increased NF-kB activity compared to normal control mice. Also, endoneurium, epineural vessels, and perineurium in sural nerve biopsies of subjects with diabetes reported activated NF-kB [81-82]. A study conducted on isolated Schwann cells concerning high glucose and low glucose media reported up-regulation of NF-kB in the hyperglycemic medium [83]. The p65 subunit of NF-kB is observed to be overexpressed in the case of acute and chronic inflammatory demyelinating polyneuropathies which indicates that NF-kB plays an important role in inflammatory demyelination [84].

Tumor Necrosis Factor-α

Tumor necrosis factor-α (TNF-α) plays an important role in the regulation of immune cells. It is up-regulated by different mediators such as lymphocytes (CD4+), activated macrophages, eosinophils, and natural killer (NK) cells. An experiment conducted to detect TNF-α involvement in diabetic neuropathy reported diabetic TNF-α (-/-) mice were protected from sensory nerve conduction velocity and motor nerve conduction velocity compared to control mice. Diabetic patients with increased TNF-α, inducible nitric oxide synthase levels have more probability to develop diabetic neuropathy [85-87]. In a study, an increase of TNF-α and monocyte chemoattractant protein-1 (MCP-1) secretion, as well as their messenger ribonucleic acid (mRNA) expression, is observed in rat microglia when treated with higher levels of glucose [88-89].

Cyclo-Oxygenase Pathway

Cyclo-oxygenase (COX) enzymes are an important part of prostaglandin synthesis and arachidonic acid metabolism. They are of two forms namely, cyclo-oxygenase 1 (COX-1) maintains cellular homeostasis and cyclo-oxygenase 2 (COX-2), upregulated by oxidative stress, protein kinase C activation, inflammatory cytokines, tumor promoters, and growth factors [90-91]. Increased COX-2 expression in the neurons of streptozotocin-induced diabetic rats has been reported. An immunity towards diabetes-induced nerve conduction deficit and decreased blood flow around myelin sheath have been reported in COX-deficient mice when compared to wild-type COX-2 (+/+) diabetic mice. This concludes that COX-2 has an important role in nerve functioning [92-93].

Other molecules and their pathways that play a prominent role include the nerve growth factor [94], lipoxygenase pathway [95-96], autophagy [97-99], wingless/integrated (Wnt) pathway [100-101], hedgehog pathway [102], and interleukins [103-105].

Central nervous system changes in diabetes

The changes seen in the brain are mainly attributed to chronic hyperglycemia and are referred to as diabetic encephalopathy [106-107]. The alteration in brain structure and function is a major concern because it has an impact on the overall quality of life. 

Brain function and structure can be affected by both acute and chronic disturbances in the vascular systems [106-108]. Hyperglycemia causes both macrovascular and microvascular complications. Cerebrovascular disease is a macrovascular complication with atherosclerosis being the main underlying cause. Hyperglycemia leads to endothelial damage through increased oxidative stress and cytokine and immune response (increased CRP, IL-6, IL-17). This leads to the formation of a thrombus that enters small vessels of the brain causing cerebral infarction [108]. This can lead to vascular cognitive impairment. Recent studies have shown plasma Kallikrein interferes with normal clotting processes in the brain following blood vessel injury due to diabetes thus causing intracerebral hemorrhage [109-110].

Several cellular processes like growth, metabolism, and differentiation need insulin/insulin-like growth factor 1 mediated activation. Insulin also plays a role in neurotransmission, synaptic plasticity and various cognitive processes, apoptosis, and antioxidant defense. Therefore, any defect in the pathways of insulin signaling can result in altered brain function [107]. T1DM causes insulin deficiency with effects on the expression of neurotrophic factors, neurotransmission, loss of functional integrity, and defects in brain connectivity. T2DM causes cognitive decline and dementia due to decreased insulin sensitivity [111-112]. Long-term diabetes has been shown to cause a decrease in insulin-like growth factors and their receptors leading to apoptosis of neurons [113-114].

The neurocognitive changes seen in diabetic patients have been associated with changes in the white and gray matter volume. These changes are particularly seen in those with long-standing hyperglycemia, early-onset disease, or recurrent episodes of severe hyperglycemia. Studies have shown a decreased volume of gray matter in the thalamus, temporal lobes, parahippocampal gyrus, insular cortex, and angular gyrus. These regions are associated with memory, attention, and language processing [14, 113]. These patients were shown to have high levels of HbA1C as well. Some structures like the cerebellum and occipital gyrus showed increased gray matter density possibly to compensate for the early retinal changes seen in diabetic patients [113, 115].

Diabetes mellitus impairs hippocampus-dependent memory through changes in hippocampal neuroplasticity. This impairs the brain’s ability to adapt and reorganize important behavioral and emotional functions [113, 116]. The hippocampus is the first region of the brain to be affected due to any kind of stress, whether it be in response to any diet, environmental factors, endocrine changes, or metabolic changes. Neuronal loss in the hippocampus is related to oxidative stress. Within the hippocampus, the most affected areas are the dentate gyrus and cornu ammunis (CA3). There is reduced dendritic spine density, synaptic proteins, and also an increase in the apoptotic markers as a result of DM. It also affects hippocampal neurogenesis (generation of new neuronal cells). Imaging shows a decreased volume of the hippocampus and electrophysiological studies reveal a reduction in long-term potentiation. This causes a decline in learning, memory, and affective expression [112, 114].

On the other hand, cognitive decline and dementia seen in diabetes are also attributed to white matter disease. Patients with T2DM are more prone to dementia than T1DM due to associated metabolic risk factors like hypertension, obesity, and hyperlipidemia [107, 117]. The white matter disease appears as hyperintensities on MRI and is due to microvascular changes in the cerebral vessels. These white matter hyperintensities were found to be larger in patients with T2DM, HbA1C >7%, and in those presenting with pre-diabetes [118]. They also cause lacunar infarcts and a decrease in white matter volume due to brain atrophy. Imagining studies have shown that DM alters the connectivity and function of white matter tracts as well. Both prediabetes and T2DM have shown a decrease in the number of white matter connections. These changes lead to poor performance in memory, attention, and executive functions [118-120]. As patients with pre-diabetes already present changes, it is important to intervene and prevent complications. 

Alzheimer’s disease (AD) due to DM is known as type 3 DM according to recent studies [116]. It has been shown that people with DM have a 65% higher chance of developing AD [121]. The cause of this is insulin resistance, imbalance in insulin growth factors, and damage to blood vessels. The accumulation of AGE products has also been implicated in the development of AD. Mitochondrial dysfunction is characterized by disruption of the electron transport chain, oxidative phosphorylation, and axonal transport which leads to synaptic dysfunction and also contributes to dementia. High levels of serum glucose have also been linked to higher beta-amyloid blocking nerve signals. Studies have shown increased beta-amyloid and neurofibrillary tangles in a diabetic brain. The brain here has a decreased ability to use and metabolize glucose [122]. It has been found that a decline in glucose processing leads to cognitive impairment, word-finding difficulty, and behavioral changes [116]. Decreased glutamate levels and N acetyl aspartate which causes loss of neuronal integrity and gliosis were also found in the brain of AD [123]. Positron emission tomography (PET) scans in AD patients demonstrated a reduction in glucose metabolism in the parietal and frontal lobes [124].

The effects on the motor system are less compared to the sensory system in DM. The motor cortex carries information of the motor commands to the brainstem nuclei and spinal motor neurons to bring a voluntary movement. Patients with long-term DM show a decrease in excitability of the motor cortex. Morphological changes of the dendritic length in the corticospinal tract and spine density have also been observed. These changes lead to decreased function of the corticospinal tract in DM due to a decrease in conduction velocities [112]. 

Effects on central nervous system, peripheral nervous system, and autonomic nervous system

Studies have recently shown that DM causes critical functional impairments notably from CNS complications [125]. Vascular and metabolic consequences of DM are significant contributors affecting the CNS and further research should conjecture the mechanism behind the CNS complications that validate these effects. Several studies have conveyed that DM is a nonpartisan risk factor for cognitive impairment [126].

The risk of cognitive impairment and microstructural alterations in white matter tracts is very high with persistently elevated blood glucose level. In adults with T2DM, cognitive dysfunction is defined as poor attention implicating work, executive function, mental processing, and recalling memory. In contrast, performances of less demanding tasks such as immediate memory and simple reaction time are not significantly altered. Additionally, lower scores on intelligence, academic accomplishments, attention, mental processing, and executive functions are observed in diabetic children and adults of T1DM. However, the mechanism behind cognitive dysfunction in T1DM is not fully understood [127]. The prevalence of both T2DM and dementia increases with age. T2DM is a significant influencing factor for dementia, especially those related to AD and there is ample evidence to support that hypothesis [128]. A recent meta-analysis revealed that T2DM has the most considerable effects on information processing speed, planning, mental efficiency, and verbal learning [129]. Brain atrophy has been illustrated in cognitively dysfunctional T2DM patients in the hippocampus and various cortical areas. High fasting blood glucose and HbA1c is associated with lower score on the Mini-Mental State Examination [130]. This report conveys the utmost importance of proper glycemic control. Epidemiological studies demonstrated that correcting metabolic factors might lessen the rate of cognitive decline and implementation of behavioral strategies is needed to increase adherence to medical regimens. Compared to the general population, a high prevalence of DM is observed in patients with involuntary movement disorders including Huntington's disease (HD), tardive dyskinesia, tremor, Parkinson's disease, and neuroleptic-induced Parkinsonism [131-133]. A research study stated that people with Parkinson's disease alone have a lower prevalence of insulin resistance than people with Parkinson’s disease with dementia [134]. Patients with HD are seven times more likely to have diabetes than the proband's non-HD relatives [135]. Amyotrophic lateral sclerosis linked copper/zinc-superoxide dismutase mutation patients have high free fatty acid levels. This is considered a significant determinant of insulin resistance predisposing neural cells to excitotoxicity.

Diabetes is responsible for 7% of deaths caused by stroke and is an established risk factor for thrombotic brain infractions of all ages [136]. The high blood glucose level at the event of cerebral ischemia exacerbates neurologic injury and even mild hyperglycemia heightens further neurologic injury and late salvage [137]. Two other lethal hyperglycemic catastrophes that transpire as acute complexities of T1DM and T2DM are conceivably diabetic ketoacidosis (DKA) and hyperglycemic hyperosmolar state (HHS), mostly affecting the CNS [138]. Disseminated intravascular coagulation and a prothrombotic state are believed to be substantial pathophysiologic contributors in DKA patients with CNS complications [139]. DKA and HHS are most likely to develop in the outpatient setting prompting hospital admission, whereas hypoglycemia is a frequent complication of glucose-lowering therapy in outpatient and inpatient. The presence of DKA, HHS, and hypoglycemia all require identifying the precipitating cause, tailoring glycemic goals, and individualizing glucose-lowering treatments according to age to prevent these potentially life-threatening diabetic complications from recurring.

Distal symmetrical sensory polyneuropathy is the most common entity of diabetic peripheral neuropathy (DPN). DPN only manifests if hyperglycemia has been present for a long-time duration. This is a length-dependent sensory-predominant process that most often rises insidiously and advances gradually. Numbness, paresthesia, or both starts in the feet and progressively ascends and dysesthetic pain is an uncommon presentation in some cases. Physical exam reveals large fiber (joint position sense, vibration sense) and small fiber (pain, temperature) sensory deficits in the feet, ankles, and hands in advanced cases. Ankle and knee reflexes might be sluggish or absent in more critical patients. Muscle atrophy and weakness are the motor symptoms, but are not present as commonly as sensory symptoms. Moreover, intrinsic muscle atrophy is mostly seen in the feet. Weakness is frequently displayed on dorsiflexion and plantarflexion of the foot with a history of balance difficulties, nighttime falls, and antalgic gait. The instability experienced by these neuropathic patients can show poor display on the tandem gait, Romberg test or one-foot stand [140]. Moreover, these people are vulnerable to infection, ulceration, burn, gan­grene, Charcot foot, and foot drop due to severe peripheral nerve involvement [141]. On the other hand, vasculopathy and higher susceptibility to compressive injury both contribute to diabetic mononeuropathies. Mononeuropathies can involve cranial nerves, nerve roots, or peripheral nerves. Peripheral mononeuropathies can occur in the arms and legs and acute diabetic femoral mononeuropathy is an archetypal diabetic mononeuropathy. Carpal tunnel syndrome can cause severe, intractable pain, debilitating hand weakness and is documented in over 30% of the diabetic population [142]. Other neuropathies include cubital tunnel syndrome and peroneal neuropathy at the fibular head. Findings such as Dupuytren’s contracture, palmar flexor tenosynovitis, and limited joint mobility are often recognized in the hands of DM patients and may add to mononeuropathy of the wrist [143]. Diabetes can also selectively damage a group of nerves in a specific region.

Chronic inflammatory demyelinating polyneuropathy (CIDP) is remarkably similar to radiculoplexus neuropathies except that radiculoplexus neuropathies are painful, whereas the former is painless. Any solid evidence that CIDP is comparatively more prevalent in the diabetic population is currently lacking. The radiculoplexus neuropathies of DM can be divided into three types: diabetic cervical radiculoplexus neuropathy (DCRPN), diabetic thoracic radicular neuropathy (DTRN), and diabetic lumbosacral radiculoplexus neuropathy (DLRPN) [144]. DLRPN (also known as diabetic amyotrophy) is known as the most prevalent type. DLRPN is commonly unilateral, asymmetric, and has a sudden onset that involves the proximal segments that quickly spread to involve the unaffected segments and the contralateral side. The most common is aching pain in the hip, buttock, thigh, leg, or foot. Although pain is the most prominent initial symptom, weakness soon follows the pain. The weakness becomes so severe that many patients start using wheelchairs at some point during their illness. Conversely, patients with DLRPN often have a better prognosis than those with diabetic sensorimotor polyneuropathy [145]. Patients who develop DCRPN acknowledge excruciating neuropathic pain, numbness, and paresthesia in the chest or abdomen followed by weakness, numbness, and atrophy in one arm [146]. It is generally considered that cranial neuropathies are higher in patients with DM when compared with the nondiabetic population and isolated thoracic radiculopathy may be confused with the prodrome of herpes zoster [147]. Patients with DM can advance to cranial neuropathy that affects the third, fourth, sixth, or seventh cranial nerve, the oculomotor nerve being the commonly affected one. Patients have unilateral ptosis, difficulty in elevation, depression, and adduction with the pupillary disturbances including the pupillomotor function damage such as the reduced diameter of the dark-adapted pupil and the Argyll-Robertson pupil. Seventh nerve palsy is another deficit with abnormal impaired glucose tolerance (IGT) test being observed in 6%-66% of cases [148]. A good number of patients who undertake quick glycemic control experience a treatment-induced sensory neuropathy (also known as insulin neuritis) which is acute and painful [149]. Patients primarily report distal sensory problems in the lower extremity.

Diabetic autonomic neuropathy (DAN) is a broader entity affecting all organs and systems in the body and presents a diverse clinical scenario. Its derivation is from endocrine factors, but it directly affects the nervous system, whose symptoms incorporate many differential diagnoses that acquaints the entire internal medicine. Clinical or laboratory characteristics of DAN are not often present when the diagnosis of DM is made because the symptoms of DAN increase with age in addition to the duration and severity of peripheral neuropathy. Subclinical autonomic dysfunction can arise within one year of diagnosis in T2DM patients and within two years in T1DM patients [150]. The prevalence of autonomic impairment is up to 54% in T1DM and 73% in T2DM patients [151]. Cardiovascular autonomic neuropathy (CAN) is the most explored and clinically crucial form of DAN with a high mortality rate. The prevalence varies from 2.5% to 50%, depending on the age period of diabetes and diagnostic criteria [152]. There is a composite association between diabetic autonomic neuropathy and hypoglycemia unawareness. A vicious cycle of hypoglycemia unawareness induces a further decline in counterregulatory hormone responses to hypoglycemia by autonomic disruption. This event occurs commonly in persons with diabetes who are in rigorous glycemic control. The malfunctioning responses can be somewhat restored by comprehensively avoiding hypoglycemia in extensively treated patients with short- and long-period diabetes [153].

The up-to-date evidence implies a range of alterable functional defects in gastrointestinal neuropathy [154]. Any section of the gastrointestinal tract could be affected with the most prevalent type being esophageal enteropathy, fecal incontinence, gastroparesis, diarrhea, and constipation. Moreover, acute onset hyperglycemia decelerates gastric emptying. Diabetic erectile dysfunction in men has a prevalence varying from 20% to >70% depending on various means [155]. According to studies, the prevalence of hypogonadism in men with T2DM varies from 20% to 60% [156]. On the contrary, female sexual dysfunction (FSD) is commonly seen in patients with T1DM [157]. Women with FSD reported a loss of libido, vaginitis, problems with orgasm, pain, decreased lubrication, and arousal. The urinary bladder is another organ affected that demonstrates dysuria, frequency, urgency, incomplete bladder emptying, nocturia, stress incontinence, and recurrent cystitis. Furthermore, there is a strong link between diabetic cystopathy and peripheral neuropathy [157]. An exclusion must be made because urological disorders such as benign prostatic hyperplasia in men and gynecological diseases in women share similar symptoms with diabetic cystopathy. Sudomotor autonomic neuropathy may result in hypo- or anhidrosis, mainly causing dryness of the foot skin that helps to form fissures, infection, and ulceration. Proper balanced glycemic control remains the foundation stone of the prevention, progression, and hindrance of DAN. In nearly all cases, symptomatic drugs such as non-steroidal anti-inflammatory drugs (NSAIDs) are the treatment of choice, although an efficient, wide-ranging pathogenetic treatment of neural decline remains to be established.

Measures to prevent and treat neuronal changes in diabetes

Considering all the above-mentioned manifestations, it is important to screen patients with DM for neurologic complications, to prevent, and to treat them. To prevent neurological alterations, the goal is strict control of serum glucose levels. There is a difference between T1DM and T2DM; in the former, there are details that improved glycemic index to prevent the development of neuronal alterations at an early stage of the disease, in contrast, there is not much evidence that this can be of benefit in T2DM [158].

A better understanding of DM and its pathophysiology are critical for patient management and prevention of further complications, which leads to better treatment adherence [159-160]. Providing education on exercise and dietary management improves glycemic levels. For meal planning, switching to a low carbohydrate diet, low-fat products, and a high fiber diet could control glucose levels. Therefore, this would slow down the progression of the disease. Some studies show that eating patterns such as the Mediterranean diet could affect glycemic levels and cardiovascular outcomes [161-162]. The ketogenic diet (KD) and caloric restriction (CR) also play a role in oxidative stress, autophagy, and signaling pathways leading to an increased insulin level, fat oxidation, decreasing adipose tissue, reducing inflammation, and improving different molecular pathways [163]. Both diets have proven benefits in epilepsy, AD, cancer, autism spectrum disorder, metabolic syndrome, vascular diseases, and other neurodegenerative disorders such as HD and PD. The KD provides ketone bodies as a brain energy source instead of glucose, stabilizing synapses and improving brain energy reserve which leads to a neuronal function enhancement. The CR also decreases glucose sensitivity, but without producing ketone bodies [163-164]. The KD should be recommended with caution, more evidence is needed to support this type of therapy, there is a concern in patients with T1DM because of the lipid profile provided by the diet, as well as in patients with T2DM with increased cardiovascular risk, therefore, each diet should be tailored for each patient [165].

Along with glycemic control, certain risk factors, such as elevated low-density lipoprotein (LDL) and cholesterol, obesity, and hypertension involved in the development of neurological alterations need to be monitored [166]. Once the neuronal alterations have started, the therapies vary depending on the progression of the disease. Therapies can target the underlying pathology or focus on relieving the symptoms [167-168]. Nevertheless, some studies show that treatments that target the underlying mechanisms have better results [169]. 

Cognitive dysfunction as a consequence of DM, should have a multidisciplinary approach considering factors such as the extent of memory impairment, age, and previous medication, with this in mind, each regimen should be tailored to each individual [170-171]. Screening for cognitive impairment should usually start at the age of 65 [172]. Management includes education to the patient and the caregiver along with care on pharmacological treatment, not to use any intensive treatment for glucose control which can lead to severe hypoglycemia and can lead to further complications [173].

Diabetic peripheral neuropathy is the most common neurologic complication seen in long-standing patients with poor control over their glucose levels. Symptomatic patients with DPN may present with foot ulcers, diabetic foot, and sometimes require amputation [167]. Nowadays, DPN is mainly treated by anticonvulsants, such as gabapentin with or without opioids, for a better effect on modulating pain [174]. In contrast, lamotrigine and sodium valproate are considered ineffective by the European Federation of Neurological Science (EFNS) [175-176]. Tricyclic antidepressants (amitriptyline is most preferred) and tetracyclic antidepressants are primarily used for neuropathic pain. Topical agents such as capsaicin (0.075%) which is a capsicum pepper extract helps to decrease mean pain intensity [177]. Some upcoming treatments target glucose metabolic pathways, such as sorbinil, an aldolase reductase inhibitor involved in the polyol pathway, is an example, but not used due to its adverse effects [178]. As aforementioned, oxidative stress plays a role in peripheral neuropathy in patients with uncontrolled DM. Nutraceutical therapies such as vitamin E can help to decrease oxidative stress as well as some dietary products such as cruciferous vegetables and red grapes [179].

However, not all therapies depend on drugs for pain relief; there is percutaneous and transcutaneous electrical nerve stimulation (PENS, TENS), and acupuncture which has a significant effect on increasing arterial circulation in patients with T2DM [180-181].

Diabetic autonomic neuropathy (DAN) affects almost all major organ systems, the treatment still relies mainly on strict glycemic control but varies depending on the system involved. In the case of cardiovascular autonomic neuropathy (CAN), alpha-lipoic acid showed some promising results in alleviating the symptoms [174]. In the case of orthostatic hypertension, management involves fluid and salt monitoring, along with physical activity [182] and if needed, pharmacologic treatment involves midodrine and droxidopa [183]. In diabetic diarrhea drugs such as loperamide are used to control the extra active bowel movements and tetracyclines are helpful to tamper the unnecessary bacterial growth [184-185]. In the case of bladder disturbance, there are no drugs that can help to alleviate symptoms, but some medications such as oxybutynin, an antimuscarinic drug, for detrusor hyperreflexia, and interventions such as intermittent self-catheterization is useful in relieving the symptoms [186]. Whereas in erectile dysfunction, statins, 5-phosphodiesterase inhibitors, and transurethral prostaglandins can be used in mild cases, however in severe cases, penile implants are preferred [187-188]. 

There are some specific treatments for T1DM such as islet transplantation which showed significant changes in the neuronal symptoms, but studies have shown that this is effective for DPN, but not quite on DAN [189]. Although the presentation of symptomatic neuronal changes in T1DM are not so obvious, spotting the sub-clinical impairment may give us the edge to prevent the progression of the disease [190].

## Conclusions

This review was aimed at understanding the various interactions between hyperglycemia and its effects on the nervous system and we have thus summarized most of the relevant literature regarding the same. The clinical burden of diabetes and diabetes-related complications is ever increasing, with risk factors like obesity witnessing an explosion globally. However, progress has been made on the therapeutic end, with a whole host of novel drugs being developed to treat the disease. Better treatment protocols, novel drugs, and recombinant insulin all help in achieving better glycemic control and thus prevent and delay the progression of neuronal changes in DM. However, there is still scope for further research regarding the same, specifically related to drugs that halt and reverse the neurological complications of DM. 

## References

[1] American Diabetes Association (2004). Diagnosis and classification of diabetes mellitus. Diabetes Care.

[2] American Diabetes Association (2010). Diagnosis and classification of diabetes mellitus. Diabetes Care.

[3] Scheen AJ (2010). Central nervous system: a conductor orchestrating metabolic regulations harmed by both hyperglycaemia and hypoglycaemia. Diabetes Metab.

[4] Seaquist ER (2015). The impact of diabetes on cerebral structure and function. Psychosom Med.

[5] Sapra A, Bhandari P (2021). Diabetes Mellitus. StatPearls.

[6] Mergenthaler P, Lindauer U, Dienel GA, Meisel A (2013). Sugar for the brain: the role of glucose in physiological and pathological brain function. Trends Neurosci.

[7] Routh VH, Hao L, Santiago AM, Sheng Z, Zhou C (2014). Hypothalamic glucose sensing: making ends meet. Front Syst Neurosci.

[8] Yoon NA, Diano S (2021). Hypothalamic glucose-sensing mechanisms. Diabetologia.

[9] Nimgampalle M, Chakravarthy H, Devanathan V (2021). Glucose metabolism in the brain: an update. Recent Dev Appl Microbiol Biochem.

[10] Vaughn AE, Deshmukh M (2008). Glucose metabolism inhibits apoptosis in neurons and cancer cells by redox inactivation of cytochrome c. Nat Cell Biol.

[11] King A, Gottlieb E (2009). Glucose metabolism and programmed cell death: an evolutionary and mechanistic perspective. Curr Opin Cell Biol.

[12] Roh E, Song DK, Kim MS (2016). Emerging role of the brain in the homeostatic regulation of energy and glucose metabolism. Exp Mol Med.

[13] Kroemer G, Mariño G, Levine B (2010). Autophagy and the integrated stress response. Mol Cell.

[14] Seaquist ER (2010). The final frontier: how does diabetes affect the brain?. Diabetes.

[15] Gispen WH, Biessels G-J (2000). Cognition and synaptic plasticity in diabetes mellitus. Trends Neurosci.

[16] Manschot SM, Biessels GJ, Rutten GE, Kessels RP, Gispen WH, Kappelle LJ (2008). Peripheral and central neurologic complications in type 2 diabetes mellitus: no association in individual patients. J Neurol Sci.

[17] Lee J, Cummings BP, Martin E (2012). Glucose sensing by gut endocrine cells and activation of the vagal afferent pathway is impaired in a rodent model of type 2 diabetes mellitus. Am J Physiol Regul Integr Comp Physiol.

[18] Vujosevic S, Muraca A, Alkabes M, Villani E, Cavarzeran F, Rossetti L, De Cillaʼ S (2019). Early microvascular and neural changes in patients with type 1 and type 2 diabetes mellitus without clinical signs of diabetic retinopathy. Retina.

[19] Xu G, Liu B, Sun Y, Du Y, Snetselaar LG, Hu FB, Bao W (2018). Prevalence of diagnosed type 1 and type 2 diabetes among US adults in 2016 and 2017: population based study. BMJ.

[20] Redondo MJ, Evans-Molina C, Steck AK, Atkinson MA, Sosenko J (2019). The influence of type 2 diabetes-associated factors on type 1 diabetes. Diabetes Care.

[21] Kennedy JM, Zochodne DW (2005). Impaired peripheral nerve regeneration in diabetes mellitus. J Peripher Nerv Syst.

[22] Grillo CA, Piroli GG, Wood GE, Reznikov LR, McEwen BS, Reagan LP (2005). Immunocytochemical analysis of synaptic proteins provides new insights into diabetes-mediated plasticity in the rat hippocampus. Neuroscience.

[23] Bulc M, Gonkowski S, Całka J (2015). Expression of cocaine and amphetamine regulated transcript (CART) in the porcine intramural neurons of stomach in the course of experimentally induced diabetes mellitus. J Mol Neurosci.

[24] Forouhi NG, Wareham NJ (2014). Epidemiology of diabetes. Medicine (Abingdon).

[25] Holman N, Knighton P, Kar P (2020). Risk factors for COVID-19-related mortality in people with type 1 and type 2 diabetes in England: a population-based cohort study. Lancet Diabetes Endocrinol.

[26] Olsson Y, Sourander P (1968). Changes in the sympathetic nervous system in diabetes mellitus. A preliminary report. J Neurovisc Relat.

[27] Watkins CC, Sawa A, Jaffrey S, Blackshaw S, Barrow RK, Snyder SH, Ferris CD (2000). Insulin restores neuronal nitric oxide synthase expression and function that is lost in diabetic gastropathy. J Clin Invest.

[28] Li C, Che LH, Ji TF, Shi L, Yu JL (2017). Effects of the TLR4 signaling pathway on apoptosis of neuronal cells in diabetes mellitus complicated with cerebral infarction in a rat model. Sci Rep.

[29] Bury JJ, Chambers A, Heath PR (2021). Type 2 diabetes mellitus-associated transcriptome alterations in cortical neurones and associated neurovascular unit cells in the ageing brain. Acta Neuropathol Commun.

[30] Sinha S, Ekka M, Sharma U, P R, Pandey RM, Jagannathan NR (2014). Assessment of changes in brain metabolites in Indian patients with type-2 diabetes mellitus using proton magnetic resonance spectroscopy. BMC Res Notes.

[31] Booya F, Bandarian F, Larijani B, Pajouhi M, Nooraei M, Lotfi J (2005). Potential risk factors for diabetic neuropathy: a case control study. BMC Neurol.

[32] Hébert HL, Veluchamy A, Torrance N, Smith BH (2017). Risk factors for neuropathic pain in diabetes mellitus. Pain.

[33] Andersen ST, Witte DR, Dalsgaard EM (2018). Risk factors for incident diabetic polyneuropathy in a cohort with screen-detected type 2 diabetes followed for 13 years: ADDITION-Denmark. Diabetes Care.

[34] Aleidan FA, Ahmad BA, Alotaibi FA, Aleesa DH, Alhefdhi NA, Badri M, Abdel Gader AG (2020). Prevalence and risk factors for diabetic peripheral neuropathy among Saudi hospitalized diabetic patients: a nested case-control study. Int J Gen Med.

[35] Dewanjee S, Das S, Das AK (2018). Molecular mechanism of diabetic neuropathy and its pharmacotherapeutic targets. Eur J Pharmacol.

[36] Bhattacharjee N, Barma S, Konwar N, Dewanjee S, Manna P (2016). Mechanistic insight of diabetic nephropathy and its pharmacotherapeutic targets: An update. Eur J Pharmacol.

[37] Brownlee M (2001). Biochemistry and molecular cell biology of diabetic complications. Nature.

[38] Tomlinson DR, Gardiner NJ (2008). Glucose neurotoxicity. Nat Rev Neurosci.

[39] Zochodne DW (2007). Diabetes mellitus and the peripheral nervous system: manifestations and mechanisms. Muscle Nerve.

[40] Zenker J, Ziegler D, Chrast R (2013). Novel pathogenic pathways in diabetic neuropathy. Trends Neurosci.

[41] Yagihashi S, Yamagishi S, Wada R (1996). Galactosemic neuropathy in transgenic mice for human aldose reductase. Diabetes.

[42] 42] Song Z (2003). Transgenic mice overexpressing aldose reductase in Schwann cells show more severe nerve conduction velocity deficit and oxidative stress under hyperglycemic stress. Mol Cell Neurosci.

[43] Kaneto H, Xu G, Song KH, Suzuma K, Bonner-Weir S, Sharma A, Weir GC (2001). Activation of the hexosamine pathway leads to deterioration of pancreatic beta-cell function through the induction of oxidative stress. J Biol Chem.

[44] Du XL, Edelstein D, Rossetti L (2000). Hyperglycemia-induced mitochondrial superoxide overproduction activates the hexosamine pathway and induces plasminogen activator inhibitor-1 expression by increasing Sp1 glycosylation. Proc Natl Acad Sci USA.

[45] Kolm-Litty V, Sauer U, Nerlich A, Lehmann R, Schleicher ED (1998). High glucose-induced transforming growth factor beta1 production is mediated by the hexosamine pathway in porcine glomerular mesangial cells. J Clin Invest.

[46] Sayeski PP, Kudlow JE (1996). Glucose metabolism to glucosamine is necessary for glucose stimulation of transforming growth factor-alpha gene transcription. J Biol Chem.

[47] Karachalias N, Babaei-Jadidi R, Ahmed N, Thornalley PJ (2003). Accumulation of fructosyl-lysine and advanced glycation end products in the kidney, retina and peripheral nerve of streptozotocin-induced diabetic rats. Biochem Soc Trans.

[48] Wada R, Yagihashi S (2005). Role of advanced glycation end products and their receptors in development of diabetic neuropathy. Ann NY Acad Sci.

[49] Tanji N, Markowitz GS, Fu C (2000). Expression of advanced glycation end products and their cellular receptor RAGE in diabetic nephropathy and nondiabetic renal disease. J Am Soc Nephrol.

[50] Ramasamy R, Yan SF, Schmidt AM (2007). Arguing for the motion: yes, RAGE is a receptor for advanced glycation endproducts. Mol Nutr Food Res.

[51] Hwang JS, Shin CH, Yang SW (2005). Clinical implications of N epsilon-(carboxymethyl)lysine, advanced glycation end product, in children and adolescents with type 1 diabetes. Diabetes Obes Metab.

[52] Sekido H, Suzuki T, Jomori T, Takeuchi M, Yabe-Nishimura C, Yagihashi S (2004). Reduced cell replication and induction of apoptosis by advanced glycation end products in rat Schwann cells. Biochem Biophys Res Commun.

[53] Vincent AM, Russell JW, Low P, Feldman EL (2004). Oxidative stress in the pathogenesis of diabetic neuropathy. Endocr Rev.

[54] Casellini CM, Vinik AI (2006). Recent advances in the treatment of diabetic neuropathy. Curr Opin Intern Med.

[55] Ko SH, Cha BY (2012). Diabetic peripheral neuropathy in type 2 diabetes mellitus in Korea. Diabetes Metab J.

[56] Edwards JF, Casellini CM, Parson HK, Obrosova IG, Yorek M, Vinik AI (2015). Role of peroxynitrite in the development of diabetic peripheral neuropathy. Diabetes Care.

[57] Obrosova I, Fathallah L, Stevens M (2002). Taurine counteracts oxidative stress and nerve growth factor deficit in early experimental diabetic neuropathy. J Peripheral Nervous Syst.

[58] Ho EC, Lam KS, Chen YS (2006). Aldose reductase-deficient mice are protected from delayed motor nerve conduction velocity, increased c-Jun NH2-terminal kinase activation, depletion of reduced glutathione, increased superoxide accumulation, and DNA damage. Diabetes.

[59] Cameron NE, Cotter MA (1994). The relationship of vascular changes to metabolic factors in diabetes mellitus and their role in the development of peripheral nerve complications. Diabetes Metab Rev.

[60] Stevens MJ, Obrosova I, Cao X, Van Huysen C, Greene DA (2000). Effects of DL-alpha-lipoic acid on peripheral nerve conduction, blood flow, energy metabolism, and oxidative stress in experimental diabetic neuropathy. Diabetes.

[61] Van Dam PS, Van Asbeck BS, Erkelens DW, Marx JJ, Gispen WH, Bravenboer B (1995). The role of oxidative stress in neuropathy and other diabetic complications. Diabetes Metab Rev.

[62] Arnoult D, Rismanchi N, Grodet A (2005). Bax/Bak-dependent release of DDP/TIMM8a promotes Drp1-mediated mitochondrial fission and mitoptosis during programmed cell death. Curr Biol.

[63] Chowdhury SK, Smith DR, Fernyhough P (2013). The role of aberrant mitochondrial bioenergetics in diabetic neuropathy. Neurobiol Dis.

[64] Borghini I, Geering K, Gjinovci A, Wollheim CB, Pralong WF (1994). In vivo phosphorylation of the Na,K-ATPase alpha subunit in sciatic nerves of control and diabetic rats: effects of protein kinase modulators. Proc Natl Acad Sci USA.

[65] Uehara K, Yamagishi S, Otsuki S, Chin S, Yagihashi S (2004). Effects of polyol pathway hyperactivity on protein kinase C activity, nociceptive peptide expression, and neuronal structure in dorsal root ganglia in diabetic mice. Diabetes.

[66] Nakamura J, Kato K, Hamada Y (1999). A protein kinase C-beta-selective inhibitor ameliorates neural dysfunction in streptozotocin-induced diabetic rats. Diabetes.

[67] Cortright RN, Azevedo JL Jr, Zhou Q, Sinha M, Pories WJ, Itani SI, Dohm GL (2000). Protein kinase C modulates insulin action in human skeletal muscle. Am J Physiol Endocrinol Metab.

[68] Szabo C, Wong H, Bauer P (1997). Regulation of components of the inflammatory response by 5-iodo-6-amino-1,2-benzopyrone, an inhibitor of poly(ADP-ribose) synthetase and pleiotropic modifier of cellular signal pathways. Int J Oncol.

[69] Homburg S, Visochek L, Moran N (2000). A fast signal-induced activation of Poly(ADP-ribose) polymerase: a novel downstream target of phospholipase c. J Cell Biol.

[70] Obrosova IG, Drel VR, Pacher P, Ilnytska O, Wang ZQ, Stevens MJ, Yorek MA (2005). Oxidative-nitrosative stress and poly(ADP-ribose) polymerase (PARP) activation in experimental diabetic neuropathy: the relation is revisited. Diabetes.

[71] Drel VR, Xu W, Zhang J (2009). Poly(ADP-ribose)polymerase inhibition counteracts cataract formation and early retinal changes in streptozotocin-diabetic rats. Invest Ophthalmol Vis Sci.

[72] Obrosova IG, Drel VR, Oltman CL, Mashtalir N, Tibrewala J, Groves JT, Yorek MA (2007). Role of nitrosative stress in early neuropathy and vascular dysfunction in streptozotocin-diabetic rats. Am J Physiol Endocrinol Metab.

[73] Das S, Joardar S, Manna P (2018). Carnosic acid, a natural diterpene, attenuates arsenic-induced hepatotoxicity via reducing oxidative stress, MAPK activation, and apoptotic cell death pathway. Oxid Med Cell Longev.

[74] Xia P, Kramer RM, King GL (1995). Identification of the mechanism for the inhibition of Na+,K(+)-adenosine triphosphatase by hyperglycemia involving activation of protein kinase C and cytosolic phospholipase A2. J Clin Invest.

[75] Xia Z, Dickens M, Raingeaud J, Davis RJ, Greenberg ME (1995). Opposing effects of ERK and JNK-p38 MAP kinases on apoptosis. Science.

[76] Dewanjee S, Joardar S, Bhattacharjee N, Dua TK, Das S, Kalita J, Manna P (2017). Edible leaf extract of Ipomoea aquatica Forssk. (Convolvulaceae) attenuates doxorubicin-induced liver injury via inhibiting oxidative impairment, MAPK activation and intrinsic pathway of apoptosis. Food Chem Toxicol.

[77] Peng G, Han M, Du Y, Lin A, Yu L, Zhang Y, Jing N (2009). SIP30 is regulated by ERK in peripheral nerve injury-induced neuropathic pain. J Biol Chem.

[78] Daulhac L, Mallet C, Courteix C (2006). Diabetes-induced mechanical hyperalgesia involves spinal mitogen-activated protein kinase activation in neurons and microglia via N-methyl-D-aspartate-dependent mechanisms. Mol Pharmacol.

[79] Purves T, Middlemas A, Agthong S, Jude EB, Boulton AJ, Fernyhough P, Tomlinson DR (2001). A role for mitogen-activated protein kinases in the etiology of diabetic neuropathy. FASEB J.

[80] Price SA, Agthong S, Middlemas AB, Tomlinson DR (2004). Mitogen-activated protein kinase p38 mediates reduced nerve conduction velocity in experimental diabetic neuropathy: interactions with aldose reductase. Diabetes.

[81] Bierhaus A, Haslbeck KM, Humpert PM (2004). Loss of pain perception in diabetes is dependent on a receptor of the immunoglobulin superfamily. J Clin Invest.

[82] Haslbeck KM, Schleicher E, Bierhaus A, Nawroth P, Haslbeck M, Neundörfer B, Heuss D (2005). The AGE/RAGE/NF-(kappa)B pathway may contribute to the pathogenesis of polyneuropathy in impaired glucose tolerance (IGT). Exp Clin Endocrinol Diabetes.

[83] Suzuki T, Sekido H, Kato N, Nakayama Y, Yabe-Nishimura C (2004). Neurotrophin-3-induced production of nerve growth factor is suppressed in Schwann cells exposed to high glucose: involvement of the polyol pathway. J Neurochem.

[84] Andorfer B, Kieseier BC, Mathey E (2001). Expression and distribution of transcription factor NF-κB and inhibitor IκB in the inflamed peripheral nervous system. J Neuroimmunol.

[85] Navarro JF, Mora C, Muros M, García J (2006). Urinary tumour necrosis factor-alpha excretion independently correlates with clinical markers of glomerular and tubulointerstitial injury in type 2 diabetic patients. Nephrol Dial Transplant.

[86] Yamakawa I, Kojima H, Terashima T (2011). Inactivation of TNF-α ameliorates diabetic neuropathy in mice. Am J Physiol Endocrinol Metab.

[87] Purwata TE (2011). High TNF-alpha plasma levels and macrophages iNOS and TNF-alpha expression as risk factors for painful diabetic neuropathy. J Pain Res.

[88] Cohn JN, Tognoni G (2001). A randomized trial of the angiotensin-receptor blocker valsartan in chronic heart failure. N Engl J Med.

[89] Quan Y, Jiang CT, Xue B, Zhu SG, Wang X (2011). High glucose stimulates TNFα and MCP-1 expression in rat microglia via ROS and NF-κB pathways. Acta Pharmacol Sin.

[90] Cosentino F, Eto M, De Paolis P (2003). High glucose causes upregulation of cyclooxygenase-2 and alters prostanoid profile in human endothelial cells: role of protein kinase C and reactive oxygen species. Circulation.

[91] Kellogg AP, Pop-Busui R (2005). Peripheral nerve dysfunction in experimental diabetes is mediated by cyclooxygenase-2 and oxidative stress. Antioxid Redox Signal.

[92] Pop-Busui R, Marinescu V, Van Huysen C (2002). Dissection of metabolic, vascular, and nerve conduction interrelationships in experimental diabetic neuropathy by cyclooxygenase inhibition and acetyl-L-carnitine administration. Diabetes.

[93] Kellogg AP, Wiggin TD, Larkin DD, Hayes JM, Stevens MJ, Pop-Busui R (2007). Protective effects of cyclooxygenase-2 gene inactivation against peripheral nerve dysfunction and intraepidermal nerve fiber loss in experimental diabetes. Diabetes.

[94] Kazanis I, Giannakopoulou M, Philippidis H, Stylianopoulou F (2004). Alterations in IGF-I, BDNF and NT-3 levels following experimental brain trauma and the effect of IGF-I administration. Exp Neurol.

[95] Obrosova IG, Stavniichuk R, Drel VR, Shevalye H, Vareniuk I, Nadler JL, Schmidt RE (2010). Different roles of 12/15-lipoxygenase in diabetic large and small fiber peripheral and autonomic neuropathies. Am J Pathol.

[96] Stavniichuk R, Drel VR, Shevalye H, Vareniuk I, Nadler JL, Obrosova IG (2010). 12/15‐lipoxygenase: roles in large and small fiber peripheral diabetic neuropathy. FASEB J.

[97] Towns R, Guo C, Shangguan Y, Hong S, Wiley JW (2008). Type 2 diabetes with neuropathy: autoantibody stimulation of autophagy via Fas. Neuroreport.

[98] Mohseni S (2011). Autophagy in insulin-induced hypoglycaemic neuropathy. Pathology.

[99] Gonzalez CD, Lee MS, Marchetti P (2011). The emerging role of autophagy in the pathophysiology of diabetes mellitus. Autophagy.

[100] Nusse R (2008). Wnt signaling and stem cell control. Cell Res.

[101] Folestad A, Ålund M, Asteberg S, Fowelin J, Aurell Y, Göthlin J, Cassuto J (2015). Role of Wnt/β-catenin and RANKL/OPG in bone healing of diabetic Charcot arthropathy patients. Acta Orthop.

[102] Chapouly C, Yao Q, Vandierdonck S, Larrieu-Lahargue F, Mariani JN, Gadeau AP, Renault MA (2016). Impaired Hedgehog signalling-induced endothelial dysfunction is sufficient to induce neuropathy: implication in diabetes. Cardiovasc Res.

[103] Bishnoi M, Bosgraaf CA, Abooj M, Zhong L, Premkumar LS (2011). Streptozotocin-induced early thermal hyperalgesia is independent of glycemic state of rats: role of transient receptor potential vanilloid 1(TRPV1) and inflammatory mediators. Mol Pain.

[104] Saleh A, Chowdhury SK, Smith DR (2013). Diabetes impairs an interleukin-1β-dependent pathway that enhances neurite outgrowth through JAK/STAT3 modulation of mitochondrial bioenergetics in adult sensory neurons. Mol Brain.

[105] Bilir B, Tulubas F, Bilir BE (2016). The association of vitamin D with inflammatory cytokines in diabetic peripheral neuropathy. J Phys Ther Sci.

[106] Mooradian AD (1988). Diabetic complications of the central nervous system. Endocr Rev.

[107] Biessels GJ, van der Heide LP, Kamal A, Bleys RLAW, Gispen WH (2002). Ageing and diabetes: implications for brain function. Eur J Pharmacol.

[108] Zhou H, Zhang X, Lu J (2014). Progress on diabetic cerebrovascular diseases. Bosn J Basic Med Sci.

[109] Liu J, Gao BB, Clermont AC (2011). Hyperglycemia-induced cerebral hematoma expansion is mediated by plasma kallikrein. Nat Med.

[110] Ergul A, Kelly-Cobbs A, Abdalla M, Fagan SC (2012). Cerebrovascular complications of diabetes: focus on stroke. Endocr Metab Immune Disord Drug Targets.

[111] Duarte JM (2015). Metabolic alterations associated to brain dysfunction in diabetes. Aging Dis.

[112] Muramatsu K (2020). Diabetes mellitus-related dysfunction of the motor system. Int J Mol Sci.

[113] Klein JP, Waxman SG (2003). The brain in diabetes: molecular changes in neurons and their implications for end-organ damage. Lancet Neurol.

[114] Foghi K, Ahmadpour S (2013). Role of neuronal apoptosis in volumetric change of hippocampus in diabetes mellitus type 1: a predictive model. ISRN Anat.

[115] Musen G, Lyoo IK, Sparks CR (2006). Effects of type 1 diabetes on gray matter density as measured by voxel-based morphometry. Diabetes.

[116] de la Monte SM, Wands JR (2008). Alzheimer's disease is type 3 diabetes-evidence reviewed. J Diabetes Sci Technol.

[117] Pourabbasi A, Tehrani-Doost M, Qavam SE, Arzaghi SM, Larijani B (2017). Association of diabetes mellitus and structural changes in the central nervous system in children and adolescents: a systematic review. J Diabetes Metab Disord.

[118] Wang DQ, Wang L, Wei MM, Xia XS, Tian XL, Cui XH, Li X (2020). Relationship between type 2 diabetes and white matter hyperintensity: a systematic review. Front Endocrinol (Lausanne).

[119] Reijmer YD, Brundel M, de Bresser J, Kappelle LJ, Leemans A, Biessels GJ (2013). Microstructural white matter abnormalities and cognitive functioning in type 2 diabetes: a diffusion tensor imaging study. Diabetes Care.

[120] Vergoossen LW, Schram MT, de Jong JJ (2020). White matter connectivity abnormalities in prediabetes and type 2 diabetes: the Maastricht study. Diabetes Care.

[121] Barbagallo M, Dominguez LJ (2014). Type 2 diabetes mellitus and Alzheimer's disease. World J Diabetes.

[122] Chatterjee S, Mudher A (2018). Alzheimer's disease and type 2 diabetes: a critical assessment of the shared pathological traits. Front Neurosci.

[123] Schuff N, Meyerhoff DJ, Mueller S, Chao L, Sacrey DT, Laxer K, Weiner MW (2006). N-acetylaspartate as a marker of neuronal injury in neurodegenerative disease. Adv Exp Med Biol.

[124] Marcus C, Mena E, Subramaniam RM (2014). Brain PET in the diagnosis of Alzheimer's disease. Clin Nucl Med.

[125] Mijnhout GS, Scheltens P, Diamant M (2006). Diabetic encephalopathy: a concept in need of a definition. Diabetologia.

[126] Allen KV, Frier BM, Strachan MW (2004). The relationship between type 2 diabetes and cognitive dysfunction: longitudinal studies and their methodological limitations. Eur J Pharmacol.

[127] Sima AA (2010). Encephalopathies: the emerging diabetic complications. Acta Diabetol.

[128] Moloney AM, Griffin RJ, Timmons S, O'Connor R, Ravid R, O'Neill C (2010). Defects in IGF-1 receptor, insulin receptor and IRS-1/2 in Alzheimer's disease indicate possible resistance to IGF-1 and insulin signalling. Neurobiol Aging.

[129] Palta P, Schneider AL, Biessels GJ, Touradji P, Hill-Briggs F (2014). Magnitude of cognitive dysfunction in adults with type 2 diabetes: a meta-analysis of six cognitive domains and the most frequently reported neuropsychological tests within domains. J Int Neuropsychol Soc.

[130] Enzinger C, Fazekas F, Matthews PM, Ropele S, Schmidt H, Smith S, Schmidt R (2005). Risk factors for progression of brain atrophy in aging: six-year follow-up of normal subjects. Neurology.

[131] Podolsky S, Leopold Norman A, Sax Danie lS (1972). Increased frequency of diabetes mellitus in patients with Huntington's chorea. Lancet.

[132] Gillman MA, Sandyk R (1986). Tardive dyskinesia and glucose metabolism. Arch Gen Psychiatry.

[133] Sandyk R (1993). The relationship between diabetes mellitus and Parkinson's disease. Int J Neurosci.

[134] Bosco D, Plastino M, Cristiano D (2012). Dementia is associated with insulin resistance in patients with Parkinson's disease. J Neurol Sci.

[135] Correia SC, Santos RX, Carvalho C (2012). Insulin signaling, glucose metabolism and mitochondria: major players in Alzheimer's disease and diabetes interrelation. Brain Res.

[136] Gray CS, French JM, Bates D, Cartlidge NE, Venables GS, James OF (1989). Increasing age, diabetes mellitus and recovery from stroke. Postgrad Med J.

[137] Kushner M, Nencini P, Reivich M (1990). Relation of hyperglycemia early in ischemic brain infarction to cerebral anatomy, metabolism, and clinical outcome. Ann Neurol.

[138] (2013). Joint British Diabetes Societies Inpatient Care Group. The management of diabetic ketoacidosis in adults. http://diabetologists-abcd.org.uk/JBDS/JBDS_IP_DKA_Adults_Revised.pdf.

[139] Timperley WR, Preston FE, Ward JD (1974). Cerebral intravascular coagulation in diabetic ketoacidosis. Lancet.

[140] Kles KA, Vinik AI (2006). Pathophysiology and treatment of diabetic peripheral neuropathy: the case for diabetic neurovascular function as an essential component. Curr Diabetes Rev.

[141] Aring AM, Jones DE, Falko JM (2005). Evaluation and prevention of diabetic neuropathy. Am Fam Phys.

[142] Dieck GS, Kelsey JL (1985). An epidemiologic study of the carpal tunnel syndrome in an adult female population. Prevent Med.

[143] Gamstedt A, Holm-Glad J, Ohlson CG, Sundström M (1993). Hand abnormalities are strongly associated with the duration of diabetes mellitus. J Intern Med.

[144] Sinnreich M, Taylor BV, Dyck PJ (2005). Diabetic neuropathies. Classification, clinical features, and pathophysiological basis. Neurologist.

[145] Dyck PJ, Windebank AJ (2002). Diabetic and nondiabetic lumbosacral radiculoplexus neuropathies: new insights into pathophysiology and treatment. Muscle Nerve.

[146] Kikta DG, Breuer AC, Wilbourn AJ (1982). Thoracic root pain in diabetes: the spectrum of clinical and electromyographic findings. Ann Neurol.

[147] Greco D, Gambina F, Pisciotta M, Abrignani M, Maggio F (2012). Clinical characteristics and associated comorbidities in diabetic patients with cranial nerve palsies. J Endocrinol Invest.

[148] Korczyn A (1971). Bell's Palsy and diabetes mellitus. Lancet.

[149] Gibbons CH, Freeman R (2015). Treatment-induced neuropathy of diabetes: an acute, iatrogenic complication of diabetes. Brain.

[150] Pfeifer MA, Weinberg CR, Cook DL, Reenan A, Halter JB, Ensinck JW, Porte D Jr (1984). Autonomic neural dysfunction in recently diagnosed diabetic subjects. Diabetes Care.

[151] Low PA, Benrud-Larson LM, Sletten DM (2004). Autonomic symptoms and diabetic neuropathy: a population-based study. Diabetes Care.

[152] Tesfaye S, Boulton AJ, Dyck PJ (2010). Diabetic neuropathies: update on definitions, diagnostic criteria, estimation of severity, and treatments. Diabetes Care.

[153] Cranston I, Lomas J, Amiel SA, Maran A, Macdonald I (1994). Restoration of hypoglycaemia awareness in patients with long-duration insulin-dependent diabetes. Lancet.

[154] Rayner CK, Horowitz M (2006). Gastrointestinal motility and glycemic control in diabetes: the chicken and the egg revisited?. J Clin Invest.

[155] Fedele D, Coscelli C, Santeusanio F (1998). Erectile dysfunction in diabetic subjects in Italy. Gruppo Italiano Studio Deficit Erettile nei Diabetici. Diabetes Care.

[156] Dhindsa S, Prabhakar S, Sethi M, Bandyopadhyay A, Chaudhuri A, Dandona P (2004). Frequent occurrence of hypogonadotropic hypogonadism in type 2 diabetes. J Clin Endocrinol Metab.

[157] Enzlin P, Rosen R, Wiegel M (2009). Sexual dysfunction in women with type 1 diabetes: long-term findings from the DCCT/ EDIC study cohort. Diabetes Care.

[158] Ohkubo Y, Kishikawa H, Araki E (1995). Intensive insulin therapy prevents the progression of diabetic microvascular complications in Japanese patients with non-insulin-dependent diabetes mellitus: a randomized prospective 6-year study. Diabetes Res Clin Pract.

[159] Rossi MC, Nicolucci A, Di Bartolo P (2010). Diabetes Interactive Diary: a new telemedicine system enabling flexible diet and insulin therapy while improving quality of life: an open-label, international, multicenter, randomized study. Diabetes Care.

[160] Bell KJ, Barclay AW, Petocz P, Colagiuri S, Brand-Miller JC (2014). Efficacy of carbohydrate counting in type 1 diabetes: a systematic review and meta-analysis. Lancet Diabetes Endocrinol.

[161] Wheeler ML, Dunbar SA, Jaacks LM, Karmally W, Mayer-Davis EJ, Wylie-Rosett J, Yancy WS Jr (2012). Macronutrients, food groups, and eating patterns in the management of diabetes: a systematic review of the literature, 2010. Diabetes Care.

[162] Bowen ME, Cavanaugh KL, Wolff K (2016). The diabetes nutrition education study randomized controlled trial: a comparative effectiveness study of approaches to nutrition in diabetes self-management education. Patient Educ Couns.

[163] Rudy L, Carmen R, Daniel R, Artemio R, Moisés RO (2020). Anticonvulsant mechanisms of the ketogenic diet and caloric restriction. Epilepsy Res.

[164] Rubio C, Luna R, Rosiles A, Rubio-Osornio M (2020). Caloric restriction and ketogenic diet therapy for epilepsy: a molecular approach involving Wnt pathway and KATP channels. Front Neurol.

[165] Bolla AM, Caretto A, Laurenzi A, Scavini M, Piemonti L (2019). Low-carb and ketogenic diets in type 1 and type 2 diabetes. Nutrients.

[166] Tesfaye S, Chaturvedi N, Eaton SE (2005). Vascular risk factors and diabetic neuropathy. N Engl J Med.

[167] Boulton AJ (2004). The diabetic foot: from art to science. The 18th Camillo Golgi lecture. Diabetologia.

[168] Adriaensen H, Plaghki L, Mathieu C, Joffroy A, Vissers K (2005). Critical review of oral drug treatments for diabetic neuropathic pain-clinical outcomes based on efficacy and safety data from placebo-controlled and direct comparative studies. Diabetes Metab Res Rev.

[169] Vollert J, Maier C, Attal N (2017). Stratifying patients with peripheral neuropathic pain based on sensory profiles: algorithm and sample size recommendations. Pain.

[170] Brown AF, Mangione CM, Saliba D, Sarkisian CA (2003). Guidelines for improving the care of the older person with diabetes mellitus. J Am Geriatr Soc.

[171] LeRoith D, Biessels GJ, Braithwaite SS (2019). Treatment of diabetes in older adults: an Endocrine Society* clinical practice guideline. J Clin Endocrinol Metab.

[172] American Diabetes Association (2019). Older adults: Standards of Medical Care in Diabetes - 2019. Diabetes Care.

[173] Sinclair AJ, Hillson R, Bayer AJ (2014). Diabetes and dementia in older people: a best clinical practice statement by a multidisciplinary National Expert Working Group. Diabetes Med.

[174] Ziegler D (2006). Treatment of diabetic polyneuropathy: update 2006. Ann NY Acad Sci.

[175] Eisenberg E, Lurie Y, Braker C, Daoud D, Ishay A (2001). Lamotrigine reduces painful diabetic neuropathy: a randomized, controlled study. Neurology.

[176] Kochar DK, Jain N, Agarwal RP, Srivastava T, Agarwal P, Gupta S (2002). Sodium valproate in the management of painful neuropathy in type 2 diabetes - a randomized placebo controlled study. Acta Neurol Scand.

[177] Tandan R, Lewis GA, Krusinski PB, Badger GB, Fries TJ (1992). Topical capsaicin in painful diabetic neuropathy. Controlled study with long-term follow-up. Diabetes Care.

[178] Judzewitsch RG, Jaspan JB, Polonsky KS (1983). Aldose reductase inhibition improves nerve conduction velocity in diabetic patients. N Engl J Med.

[179] Triantafillidis JK, Kottaras G, Sgourous S (1998). A-beta-lipoproteinemia: clinical and laboratory features, therapeutic manipulations, and follow-up study of three members of a Greek family. J Clin Gastroenterol.

[180] Kumar D, Marshall HJ (1997). Diabetic peripheral neuropathy: amelioration of pain with transcutaneous electrostimulation. Diabetes Care.

[181] Abuaisha BB, Costanzi JB, Boulton AJM (1998). Acupuncture for the treatment of chronic painful peripheral diabetic neuropathy: a long-term study. Diabetes Res Clin Pract.

[182] Freeman R, Abuzinadah AR, Gibbons C, Jones P, Miglis MG, Sinn DI (2018). Orthostatic hypotension : JACC state-of-the-art review. J Am Coll Cardiol.

[183] Kaufmann H (2017). Droxidopa for symptomatic neurogenic orthostatic hypotension: what can we learn?. Clin Auton Res.

[184] Ogbonnaya KI, Arem R (1990). Diabetic diarrhea. Pathophysiology, diagnosis, and management. Arch Intern Med.

[185] Murao S, Hosokawa H (2010). Serotonin 5-HT3 receptor antagonist for treatment of severe diabetic diarrhea. Diabetes Care.

[186] Pop-Busui R, Boulton AJ, Feldman EL (2017). Diabetic neuropathy: a position statement by the American Diabetes Association. Diabetes Care.

[187] Hackett G, Kirby M, Wylie K, Heald A, Ossei-Gerning N, Edwards D, Muneer A (2018). British Society for Sexual Medicine Guidelines on the management of erectile dysfunction in men - 2017. J Sex Med.

[188] Mulhall JP, Giraldi A, Hackett G (2018). The 2018 revision to the process of care model for management of erectile dysfunction. J Sex Med.

[189] Navarro X, Sutherland DE, Kennedy WR (1997). Long-term effects of pancreatic transplantation on diabetic neuropathy. Ann Neurol.

[190] Karavanaki K, Baum JD (2003). Coexistence of impaired indices of autonomic neuropathy and diabetic nephropathy in a cohort of children with type 1 diabetes mellitus. J Pediatr Endocrinol Metab.

